# Perceptions of Undergraduate Ophthalmology Teaching: A Survey of Medical Students in the United Kingdom

**DOI:** 10.7759/cureus.117476

**Published:** 2026-10-05

**Authors:** Omar Hamdan, Humzah Hameed, Muhammad Hamza Ahmed, Sulayman Khan, Abutorab Islam, Yaseen Elhowairis, Zayd Mazhar, Tanzil Islam

**Affiliations:** 1 Ophthalmology, University College Hospital, London, GBR; 2 Cardiology, University College London, London, GBR; 3 Ophthalmology, University College London, London, GBR; 4 Ophthalmology, Guy's Hospital, London, GBR; 5 Urology, University College London, London, GBR; 6 Dentistry, Ipswich Hospital, London, GBR; 7 Urology, Bedford Hospital, London, GBR

**Keywords:** clinical placement, confidence and competence, medical education, medical training gaps, ophthalmology education, ophthalmology teaching, red flag recognition, survey study, uk medical schools, undergraduate curriculum

## Abstract

Background: In order to prepare future medical professionals to diagnose and treat common and urgent eye disorders, adequate undergraduate ophthalmology education is essential. This study aims to analyse the confidence and ability of medical students in the United Kingdom (UK) and resident doctors in identifying red flag ophthalmological symptoms, as well as their impressions of their ophthalmology education.

Methods: Between July and September 2024, UK medical students of all years and resident doctors responded to an anonymous online survey. University officials and medical school group chats were used to recruit participants. Data on exposure to ophthalmology placement, perceived education quality and awareness of urgent ophthalmological diseases were gathered through the survey. Responses were analysed using the Qualtrics platform (Qualtrics, LLC, Provo, UT, USA).

Results: A total of 300 respondents from 23 medical schools took part in the survey, including 211 final-year students. Of those surveyed, 32.7% said they would not obtain any ophthalmology placements during their medical degree. 54.9% of respondents said they wished their medical school offered more ophthalmology instruction. Just 37.2% of respondents correctly identified all conditions requiring referral within 24 hours, indicating a low level of competence in detecting urgent referral problems. Additionally, a sizable percentage of students stated that they had not received enough instruction in fundamental abilities, including fundoscopy and slit lamp examination.

Conclusions: Our research reveals notable discrepancies in undergraduate ophthalmology instruction across UK medical schools, which contribute to students' lack of confidence and proficiency in identifying and handling ophthalmological emergencies. We have formalised some recommendations, from proven examples at other institutions, to address these shortcomings and enhance patient care outcomes. Medical schools should increase the availability of ophthalmology placements and electives and move towards blended, interactive teaching such as problem-based learning and technology-enhanced resources. National standardisation of core competencies, such as fundoscopy, slit-lamp examination and red-flag recognition, could help ensure consistent ophthalmology training across the UK.

## Introduction

The General Medical Council's (GMC) Outcome for Graduates document outlines the knowledge, skills and behaviours expected of medical graduates in the United Kingdom (UK), emphasising the diagnosis and management of conditions [1]. The 2020 Royal College of Ophthalmologists (RCOphth) Curriculum for Undergraduate and Foundation Doctors further outlines the knowledge required for the diagnosis and management of chronic and emergency eye conditions [2]. Despite these outlines, GMC requirements for ophthalmology placements dictate that not all medical schools need to provide one. With 1.5%-6% of accident and emergency (A&E) presentations and 1%-2% of general practice (GP) presentations being eye-related issues, ensuring the confidence and competency of new graduates in dealing with common and emergency ophthalmology presentations is essential [3, 4]. The projected increase in acute and outpatient ophthalmology consultations due to an ageing population and increasing comorbidities makes this issue particularly pertinent [5].

Previous literature has detailed the shortcomings of the current undergraduate ophthalmology teaching, with a reduction in quantity and length of clinical placements being offered to medical students [6]. All medical schools surveyed by Baylis et al. had covered the emergency eye presentations as part of their curriculum, but despite this, many medical students and foundation doctors have expressed their lack of confidence in dealing with them [6,7]. Numerous studies quantify the problem, with one finding that 37% of foundation doctors express that they do not feel confident in examining the visual system, and a further 39% feel they have insufficient knowledge [6]. Another study showed over half of clinical year medical students surveyed voiced similar concerns, as well as a lack of fundoscopy and slit lamp skills teaching [6-8].

This study has the primary aim to investigate the perceptions of UK medical students and foundation doctors regarding their ophthalmology teaching experiences, as well as assess their confidence and competence in red flag symptom recognition. 

The research questions of this study were as follows: Are there differences in ophthalmology teaching and exposure across medical schools in the UK? How confident and competent are clinical year medical students in red flag recognition and management?

## Materials and methods

Study design and participants

A cross-sectional online survey was conducted using Qualtrics (Qualtrics, LLC, Provo, UT, USA). According to the survey response timestamps, data were collected between the 31^st^ of July and the 20^th^ of September 2024. Eligible participants were medical students enrolled at UK medical schools in any year of study and doctors undertaking Foundation Year 1 (FY1) or Foundation Year 2 (FY2) training in the UK.

Participants were recruited using non-probability convenience and snowball sampling. A standardised invitation containing a link to the survey was circulated through medical-school WhatsApp year-group chats (Meta Platforms, Menlo Park, CA, USA) and via student representatives at universities across the UK. Recipients were able to forward the invitation and survey link to other eligible participants. Student representatives were recruited by email. Responses were received from students at 23 UK medical schools.

Participation was voluntary, and no incentives were offered. A reminder was circulated two weeks before the survey closed. As recruitment was conducted through group messaging networks and onwards forwarding of the survey link was permitted, the total number of eligible individuals who received or viewed the invitation could not be determined. Consequently, a conventional response rate could not be calculated.

Survey development and content

The questionnaire comprised 13 substantive question prompts, together with an optional field asking participants to identify the person who had referred them to the survey (Appendices). Several questions contained multiple statements or response items. Based on recorded completion times, the questionnaire took approximately three minutes to complete.

Questions collected information on participants' medical school and stage of training; exposure to ophthalmology through scheduled clinical placements and Student-Selected Components (SSCs); perceptions of the adequacy of undergraduate ophthalmology education; confidence in diagnosing and managing common ophthalmological presentations, performing basic ophthalmoscopy and making appropriate referrals; recognition of ophthalmological presentations requiring urgent assessment; and knowledge of, perceptions of and interest in the UK ophthalmology training pathway.

Participants were asked whether they had been, or expected to be, offered a clinical ophthalmology placement, the duration of any placement and whether an ophthalmology-related SSC was available at their medical school. They were also asked about the adequacy of ophthalmology teaching and clinical exposure and which additional educational opportunities they considered most beneficial.

Perceptions and self-reported confidence were assessed using 5-point Likert scales ranging from “strongly disagree” to “strongly agree”. Questions relating to ophthalmology as a career assessed participants' interest in surgery, awareness of portfolio requirements for ophthalmology specialty training, perceptions of the competitiveness and work-life balance of ophthalmology training, and perceptions of gender and racial diversity within the ophthalmology workforce.

Recognition of ophthalmological presentations requiring urgent assessment was evaluated using a multiple-response question asking participants which of eight listed presentations they believed would require urgent referral to the ophthalmology team within 24 hours. A response was classified as correct only if the participant selected all presentations designated as requiring same-day assessment.

Ethical considerations

Before beginning the survey, participants were provided with information about the purpose of the study, the voluntary nature of participation, the intended use of their responses and the handling of study data. Proceeding to complete the questionnaire was taken to indicate informed consent.

Survey data were stored on the Qualtrics platform and were accessible only to members of the study team.

## Results

Demographics

A total of 300 UK medical students and resident doctors responded to the survey, from 23 different medical schools (Figure 1). Of all respondents, 211 were final-year medical students; 196 answered all questions from the survey.

**Figure 1:**
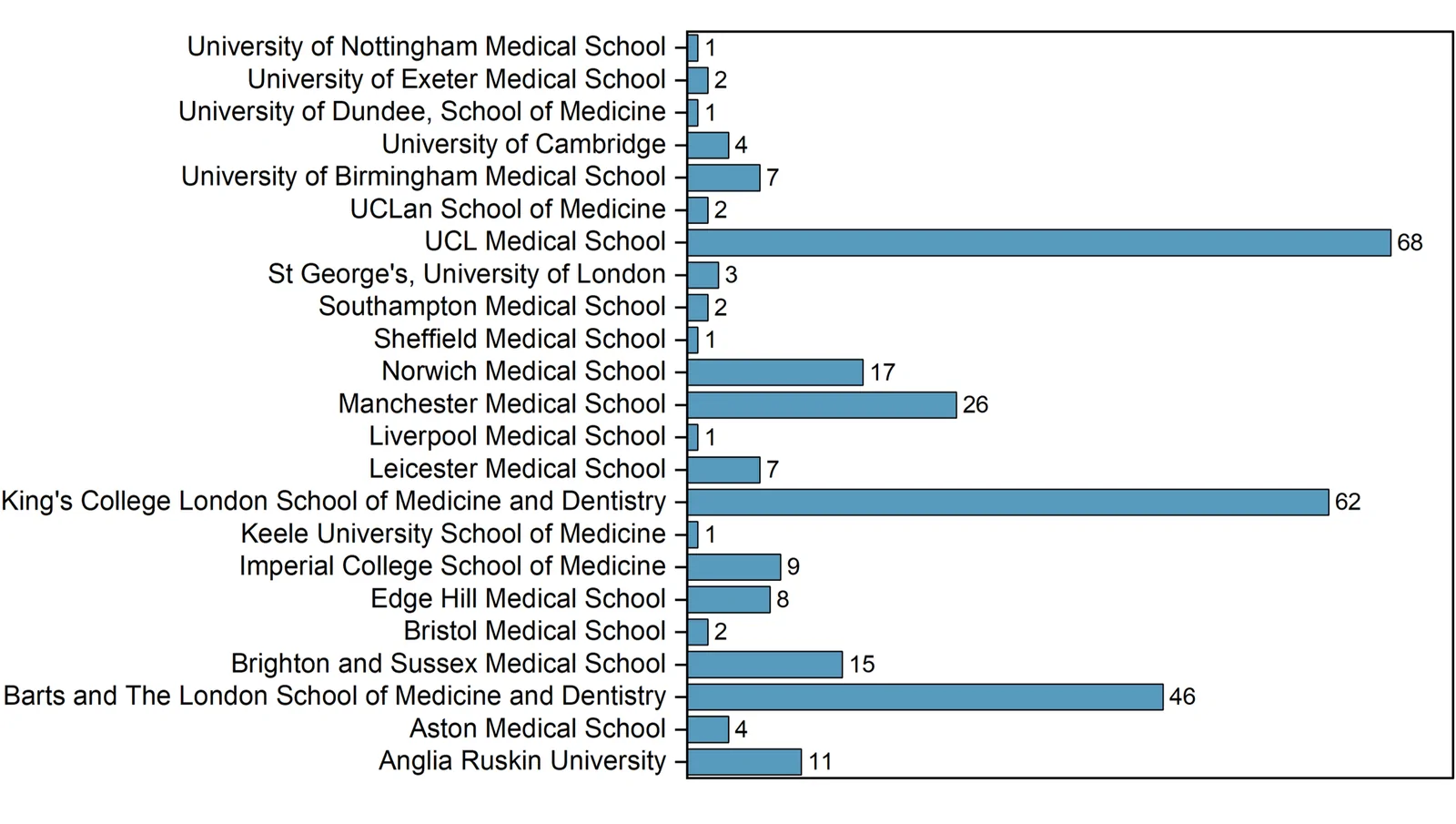
Number of respondents from each of the 23 participating UK medical schools. Data are presented as frequency (n); N = 300 respondents (medical students and resident doctors).

Teaching and exposure

Of the 300 respondents, 128 (42.7%) reported that they had or would have ophthalmology as part of their clinical placements, 98 (32.7%) indicated they would not, and 74 (24.7%) were unsure (Figure 2). Additionally, 47 respondents (15.7%) reported that ophthalmology was offered as an SSC at their medical school, 56 (18.7%) reported it was not, and 197 (65.7%) were unsure (Figure 3).

**Figure 2:**
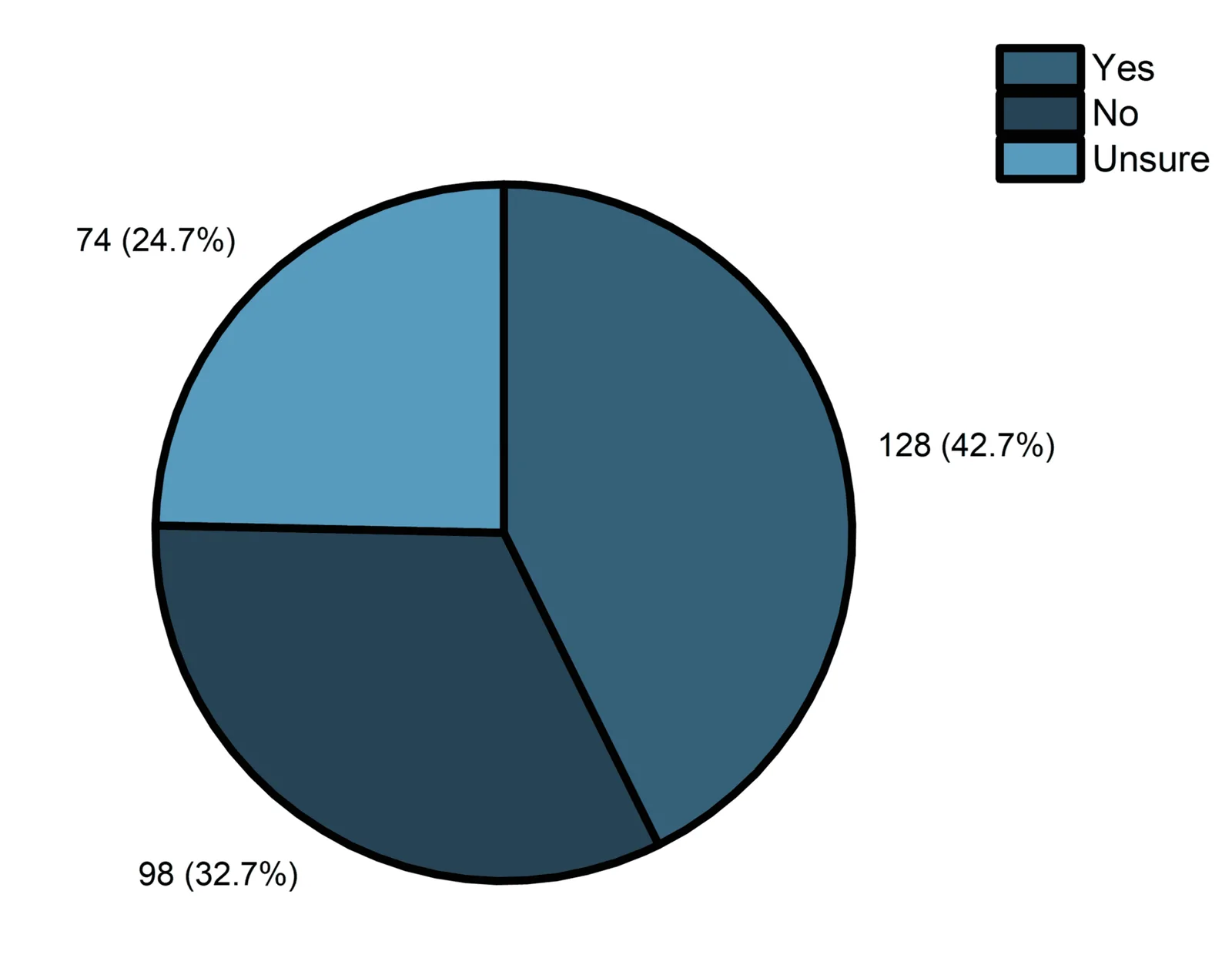
Access to ophthalmology clinical placements during undergraduate training. Responses are presented as n (%); N = 300 valid responses.

**Figure 3:**
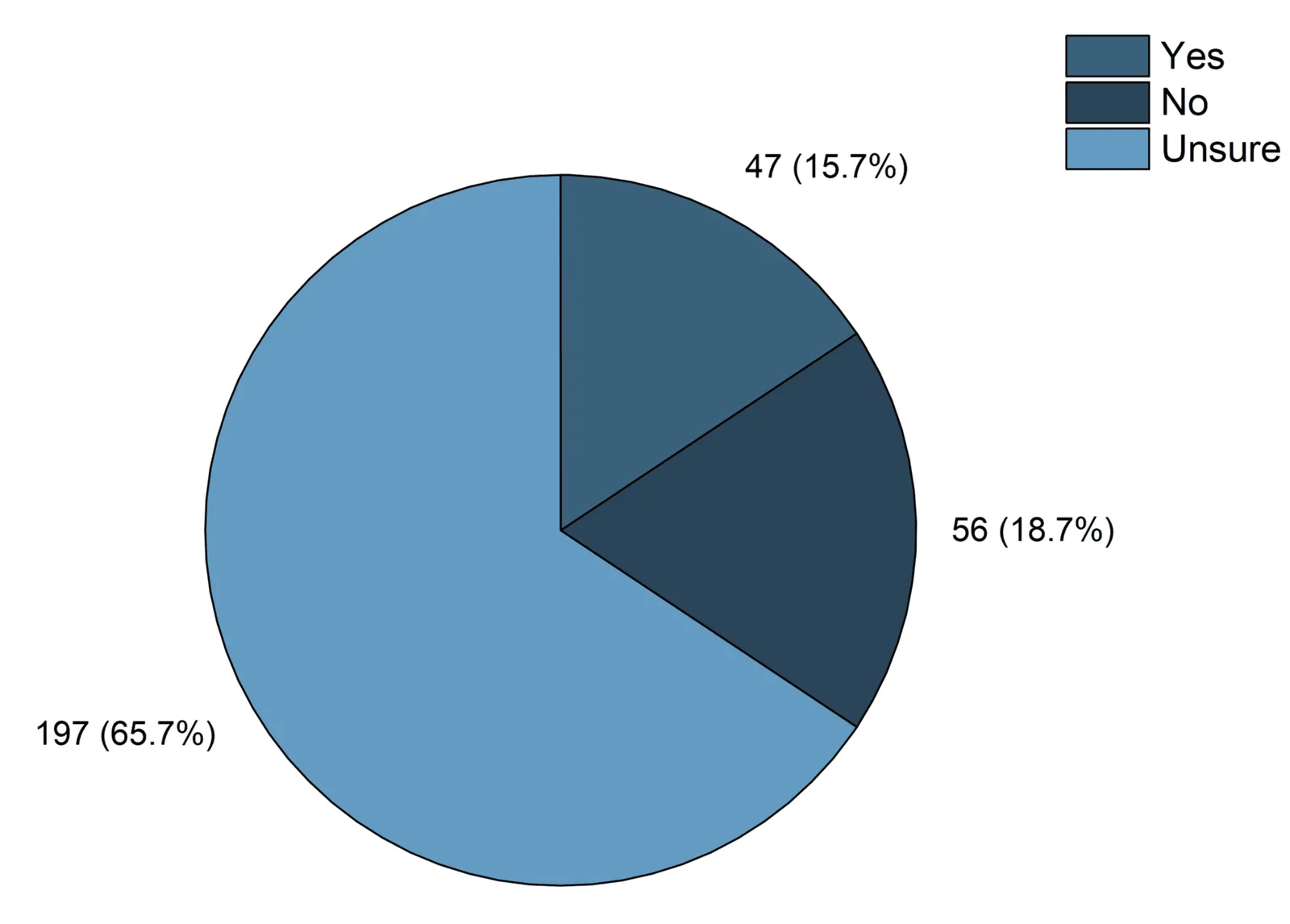
Availability of ophthalmology Student-Selected Components. Responses are presented as n (%); N = 300 valid responses.

Among the 98 respondents who indicated they had not or would not have an ophthalmology clinical placement, 32 (32.7%) reported not being offered ophthalmology as part of the SSC, and 53 (54.1%) were unsure about its availability (Figure 4).

**Figure 4:**
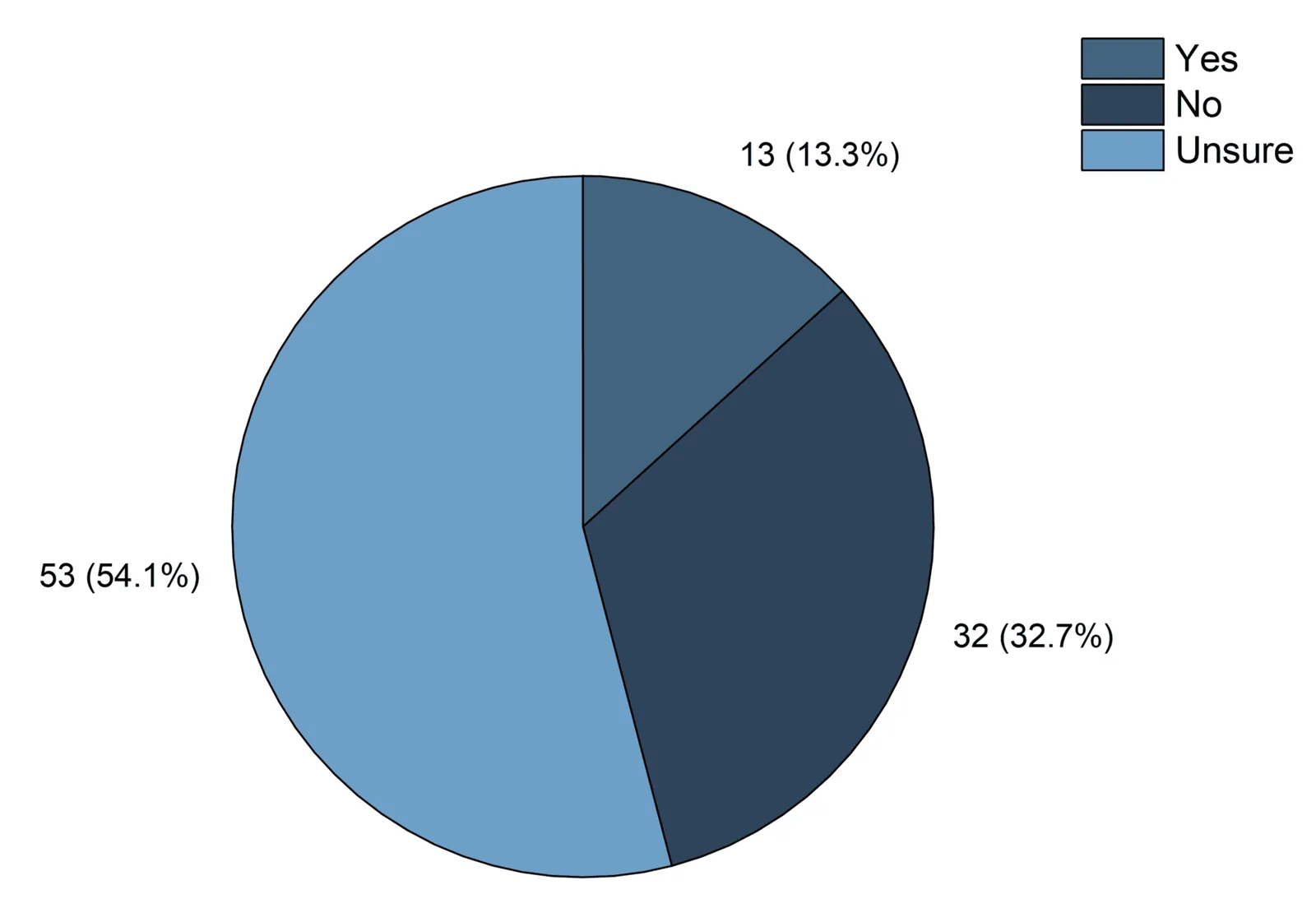
Availability of ophthalmology student selected components among respondents without a clinical placement. Responses are presented as n (%); N = 98 valid responses.

In response to the statement that their medical school provided adequate ophthalmology teaching and placement, 57 respondents (59.4%) neither agreed nor disagreed. A further 30 respondents (31.3%) disagreed or strongly disagreed, whereas only nine (9.4%) agreed or strongly agreed. In contrast, 64 respondents (66.7%) agreed or strongly agreed that medical students at their university would benefit from more ophthalmology teaching and placement. Only six respondents (6.3%) disagreed or strongly disagreed with this statement, while 26 (27.1%) neither agreed nor disagreed (Figure 5).

**Figure 5:**
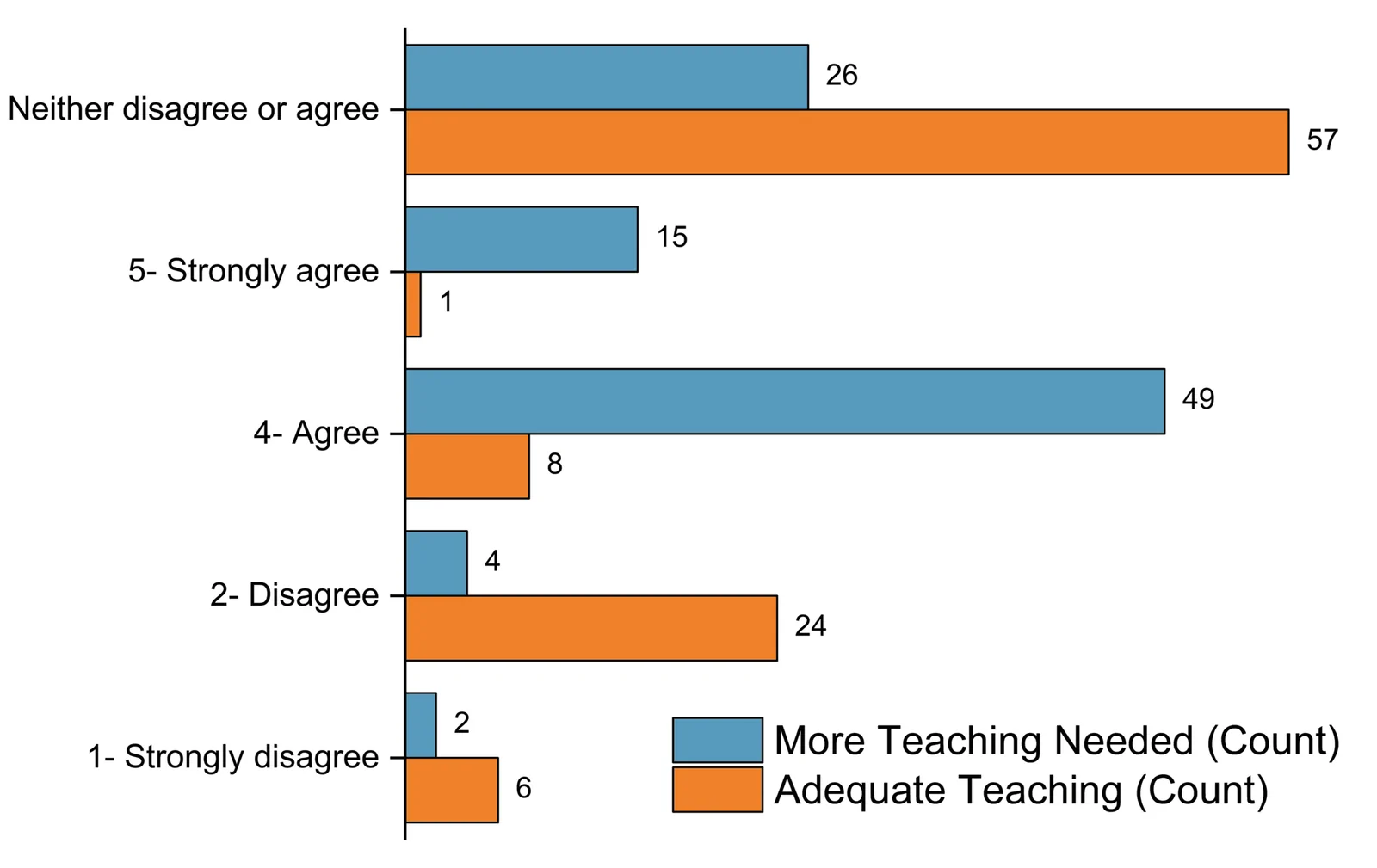
Perceptions of ophthalmology teaching and placement, rated on a 5-point Likert scale. Responses are presented as frequency (n); N = 96 valid responses.

Knowledge of ophthalmology

Of the 196 respondents, only 73 (37.2%) correctly identified that all the listed conditions required urgent referral to ophthalmology within 24 hours (Figure 6). Correct identification of all urgent presentations increased with stage of training: 14 of 54 preclinical students (25.9%), 42 of 114 clinical students (36.8%), and 16 of 25 FY1 and FY2 doctors (64.0%) (Figure 7).

**Figure 6:**
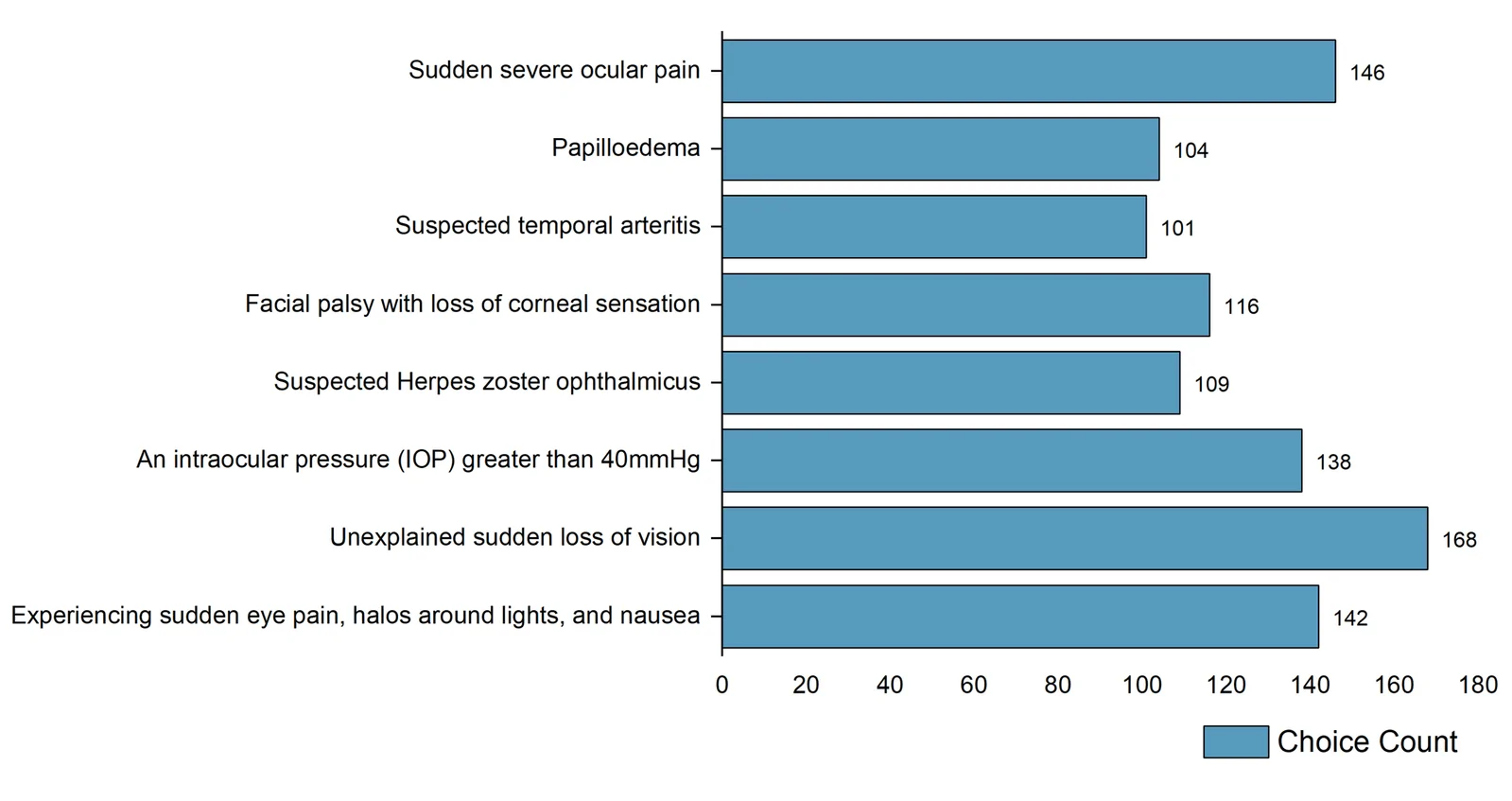
Recognition of ophthalmic presentations requiring urgent referral. Responses are presented as frequency (n), with multiple selections permitted; N = 196 valid responses.

**Figure 7:**
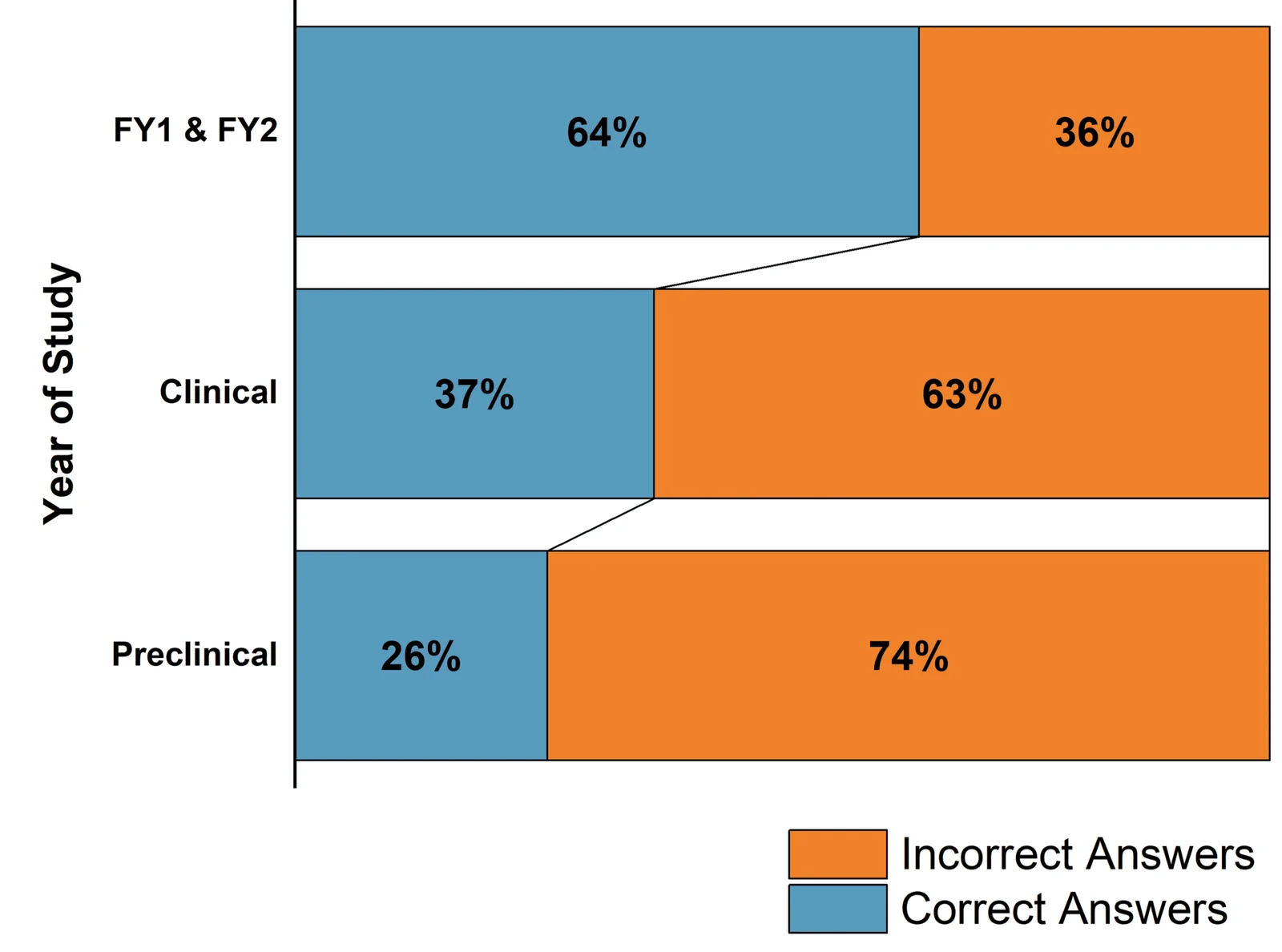
Correct identification of urgent ophthalmic referrals by stage of training. Responses are presented as percentages (%); N = 193 valid responses (preclinical, n = 54; clinical, n = 114; FY1 and FY2, n = 25). FY1: Foundation Year 1; FY2: Foundation Year 2.

Confidence in referral 

Confidence in knowing when to refer to ophthalmology similarly increased with stage of training. Of the 206 respondents, five of 63 preclinical students (7.9%), 46 of 118 clinical students (39.0%), and 14 of 25 FY1 and FY2 doctors (56.0%) agreed or strongly agreed with the statement 'I am confident in knowing when a referral to ophthalmology is needed' (Figure 8).

**Figure 8:**
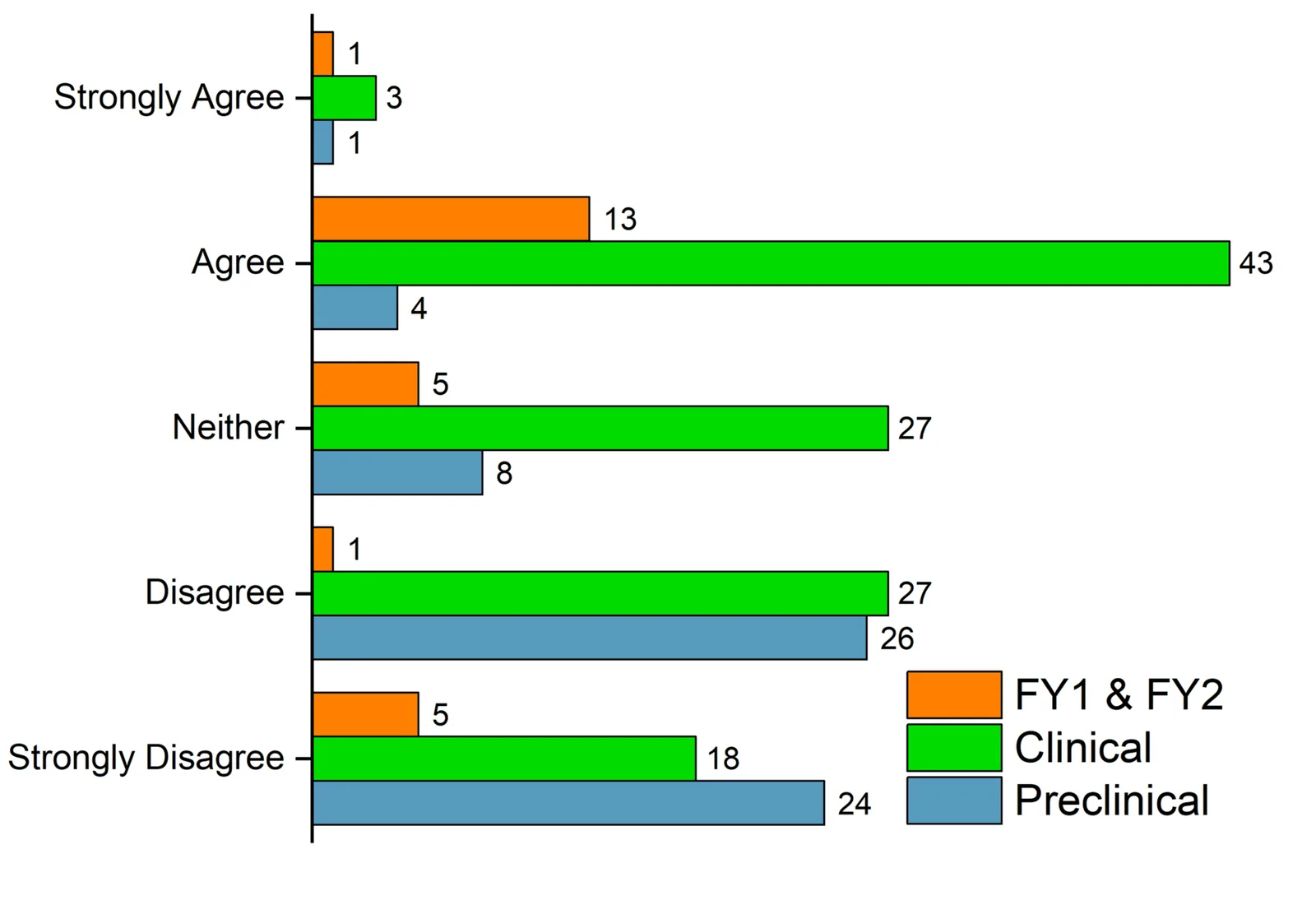
Confidence in identifying the need for ophthalmology referral by stage of training, rated on a 5-point Likert scale. Responses are presented as frequency (n); N = 206 valid responses (preclinical, n = 63; clinical, n = 118; FY1 and FY2, n = 25). FY1: Foundation Year 1; FY2: Foundation Year 2.

## Discussion

Is there a lack of ophthalmology teaching in the UK, and is this related to issues with care?

From our survey, we were able to assess the ophthalmology exposure of 23 medical schools within the UK out of 46. The survey results revealed that fewer than half of respondents (128/300, 42.7%) had or expected to have an ophthalmology placement during medical school (Figure 2). This highlights inconsistencies in how ophthalmology is integrated into the medical curriculum across different schools. Kumar et al. (2016) conducted trials to assess the reasons for missed referrals of patients with visual impairment [9]. The results identified that 39% of missed referrals were due to “clear misses and non-referrals by the physician”. The study further suggested that increased training needs to be delivered in medical schools in order to reduce the likelihood of this event occurring [9]. From this study alongside our results, we can see that undergraduate ophthalmology education is inconsistent nationally, which can subsequently affect quality of care provided to patients and ultimately their quality of life.

There is currently an increase in stress on the eye casualty services within the UK [6]. From our results, we believe that the lack of ophthalmology support within the medical school system has possibly contributed to this. Siempis et al. mentioned in their paper that the majority of GP referrals were due to “insufficient clinical skills or ophthalmic knowledge”, which increased the number of patients using the eye casualty service who in fact did not need it [10]. Furthermore, it was a similar case with A&E referrals, and it was found that having slit-lamp training for the A&E Senior House Officers (SHOs) increased their confidence by 11.5% [10]. We can therefore theorise that ophthalmology has been a neglected speciality within medical schools for decades and simple measures can be made for improvement.

A study conducted on medical students enrolled in the Royal College of Surgeons in Ireland further displayed the gap in ophthalmology knowledge among medical students [11]. As a result, they conducted a trial where multiple methods of teaching were incorporated into their curriculum. The control group was medical students who only had access to online ophthalmology teaching versus students who attended on-site and online teaching sessions accompanied by in-person patient-centred clinical skills teaching. The results showed that 65% of the test group felt that the course significantly improved their analytical skills against 43% in the control [11]. This gap in availability highlights a need for more ophthalmology focus within medical schools.

Our study shows around a third of respondents disagreed or strongly disagreed that their medical school provided adequate ophthalmology teaching, and 59.4% (57/96) selected a neutral position (Figure 5). This suggests that many students do not feel their education in ophthalmology is sufficient, particularly in practical skills such as fundoscopy and slit lamp use. The lack of objective assessments in these areas may be contributing to the students' lack of confidence. The Canadian Ophthalmology Society conducted a study on the importance of competency-based medical education (CBME) [12]. This is where they had assessments based on clinical skills, such as slit lamps, fundoscopy, etc., and as a result led to students gaining increased practice and experience to identify pathology [12]. From their results, they concluded that there was widespread incompetence with regard to practical skills, and as a result, they have incorporated CBME early in the medical programme [12].

In a study conducted with 1,070 students in 2019 assessing ophthalmology teaching in undergraduates within the UK medical school system, 75% of respondents felt that their ophthalmology teaching was insufficient and 52% did not feel confident in their ophthalmology knowledge [6]. Furthermore, an additional study was conducted questioning the teaching leads of 31 medical schools across the UK on their ophthalmology teaching. It was concluded from this study that although the teaching requirements match the RCOphth guidelines, it isn’t consistent with the International Council of Ophthalmology (ICO) [13]. These two studies are consistent with our results and allow us to suggest an increased focus on ophthalmology-specific teaching within medical schools.

How confident and competent are medical students and foundation year doctors in red flag recognition and management?

Our study evaluated preclinical medical students, clinical medical students and foundation year doctors’ confidence and competency in red flag recognition.

Confidence

When asked to what extent participants agreed with the statement: ‘With my current knowledge I can diagnose and treat common ophthalmic conditions’, only 25.2% (30/119) of clinical year medical students and 28% (7/25) of foundation doctors agreed, echoing similar findings with another typically short medical school placement, ENT [14]. Interestingly, none of the respondents strongly agreed with the statement, indicating an overall lack of confidence in diagnosing and treating common ophthalmic conditions. Furthermore, when asked the same question with the following prompt: ‘I can perform basic ophthalmoscopy and identify common abnormalities’, 32.8% (39/119) of clinical year medical students and 20% (5/25) foundation year doctors agreed. Tariq et al. report similar findings, with a significant portion of students in clinical years (52%) expressing concerns about their ability to perform fundoscopy and use a slit lamp [6]. This, along with the finding that 39% of foundation doctors feel they have insufficient ophthalmology knowledge [6], demonstrates that foundation doctors continue to feel unprepared even after medical school.

Our final prompt of: ‘I am confident in knowing when a referral to ophthalmology is needed’, had 39% (46/118) of clinical medical students and 56% of foundation year doctors in agreement (Figure 8). This sentiment is particularly concerning for two reasons, the first being the common foundation doctor job of clerking in A&E, which has a reported 1.5-6% proportion of ophthalmology presentations [3;4], and the role of a foundation year doctor in care escalation and referrals.

Competence

Our study found that 25.9% of preclinical medical students, 36.8% of clinical medical students, and 64% of foundation year doctors correctly identified all the listed conditions detailed by the 2020 RCOphth Curriculum for Undergraduate and Foundation Doctors requiring urgent referral to ophthalmology within 24 hours [2]. Other studies show that confidence in recognition and management of emergency conditions in different specialties is generally better compared to our findings [15]. One study, for example, demonstrates high levels of medical student confidence and competence in ST-elevation myocardial infarction (STEMI) recognition and initial management [15]. This highlights a gap in red flag recognition, confidence and competence in ophthalmology conditions, which is especially significant given the projected increase in acute ophthalmology presentations due to an ageing population [5].

Limitations

Despite our best efforts, our study had a number of limitations. Firstly, our responses were collected from 23 different medical schools, each with different representation in quantity, and all varied in teaching approaches and structure of clinical placements. As a result, some universities may have offered clinical ophthalmology as part of a different specialty attachment, which was unable to be recorded. Also, our survey was distributed via WhatsApp, through a network of university representatives of this study. This excludes participants who may not have access to these groups, potentially leading to selection bias in participation. Furthermore, due to the cross-sectional nature of this survey, we were unable to assess the long-term changes in ophthalmological knowledge with regard to red flags and skills confidence and competence, and whether these had tangible impacts once participants started practice as foundation doctors.

As participation was voluntary, respondents may have included a higher proportion of students with an interest in ophthalmology or its teaching. The use of snowball sampling allowed wide reach across UK medical schools, although it meant a formal response rate could not be calculated and representation varied between institutions. The findings should therefore be read as an indication of students' experiences rather than a fully representative national picture, and they provide a strong basis for larger, more structured studies in the future.

Future work

Evaluation of undergraduate ophthalmology teaching would benefit from discussion with a variety of stakeholders including medical schools, hospital placement providers, and clinical year medical students. Additionally, an evaluation of postgraduate ophthalmology teaching would be beneficial in identifying commonly missed undergraduate curriculum points.

Recommendations

Our findings suggest that medical schools should increase the visibility and availability of ophthalmology placements and electives to ensure all students receive adequate exposure to this specialty. In our study, 32 respondents (10.7%) reported that no ophthalmology student-selected component was offered at their medical school (Figure 4). Research from The University of Pittsburgh School of Medicine demonstrated that a one-week interactive ophthalmology placement significantly increased students' confidence in using ophthalmic equipment, understanding terminology, and managing diagnoses [16]; 83% of 64 participating students strongly agreed or agreed that “the overall teaching in ophthalmology clinical settings was of high quality,” and over 70% of the students achieved above 80% in a written ophthalmology examination [16]. This highlights the critical role of integrating ophthalmology education within medical curricula, as it enables students to develop familiarity with ophthalmic equipment, diagnostic processes, and patient management, ultimately addressing the current lack of confidence many medical students feel in handling ophthalmology cases.

Medical schools should also consider adapting their teaching methods to a blended learning approach, combining traditional in-person learning experiences with digital or online educational resources. A study by the Royal College of Surgeons in Ireland demonstrated that a blended learning approach, which increased student interaction with ophthalmology patients and teaching, led to a statistically significant rise in student satisfaction, confidence, and problem-solving skills within the field [11]. Additionally, a shift to more interactive teaching methods, such as problem-based learning (PBL), could be beneficial. In PBL, students learn by working through real or simulated clinical cases rather than relying solely on self-directed learning (SDL). In Saudi Arabia, Atta and Alghamdi compared SDL and PBL in a cohort of students, finding SDL less effective for promoting self-readiness [17]. A greater proportion of students from this cohort scored high to moderate marks in the PBL portion of their ophthalmology exams compared to the SDL section [17,18]. This emphasises the need for medical schools to shift away from traditional, seminar-based and textbook-based approaches to more interactive, problem-solving-based approaches, allowing students to build clinical skills through applied learning.

In addition to a blended learning approach, medical schools could use technology-enhanced learning (TEL) to improve the quality of teaching. TEL refers to the use of technology to support and enrich educational experiences, such as through virtual models, videos, and presentations. In Canada, Steedman et al. designed multimedia learning tools (MLTs) in the form of educational presentations consisting of audio, video, text, and visual images and found that students who used these achieved higher scores in questionnaires and spent 72% less time reviewing content than those using textbook excerpts of the same content [19]. Similarly in the UK, a group of students at Sussex Eye Hospital was taught ocular anatomy using a 3D biomedical visualisation software, and it was reported that 70% of the students would recommend its future use in teaching [20]. Thus, incorporating TEL into blended learning can enhance student engagement while reducing study time. However, there is a notable limitation to note on the scalability and large expense associated with having such technology readily available for all medical students across the UK.

The Objective Assessment of Skills in Intraocular Surgery (OASIS) tool, developed at Harvard Medical School, was designed to assess residents in their surgery competency [21]. Although the tool was used during residency, important lessons can be taken from this implementation and could potentially be utilised within medical schools. The OASIS programme worked by delivering pre-, concurrent and post-procedure in-depth teaching, exposing the students to a wide variety of learning types such as theory, placement and simulation [22]. A study conducted to see the effectiveness of this approach showed that the incidence of complications in the post-OASIS group was statistically significantly reduced (1.4% versus 7.7%, p < 0.0001) [22]. Thus, implementing assessments centred around ophthalmology can allow focus on core skills such as fundoscopy, slit lamp examinations and red flag recognition, to ensure that students are developing the practical skills they need to handle real-world scenarios confidently.

Finally, national standardisation of ophthalmology education could help ensure all medical students receive comprehensive and consistent training [16]. Collaboration between the GMC, the RCOphth and medical schools can lead to the development of standardised competencies and minimum clinical exposure requirements, ensuring that ophthalmology education is consistent across the UK.

## Conclusions

These findings suggest that current undergraduate provision may not adequately prepare graduates for the eye presentations they will encounter in emergency and GP settings. Increasing the availability of ophthalmology placements, adopting blended and interactive teaching methods, and introducing structured assessment of core skills such as fundoscopy and red-flag recognition could help address these gaps. National standardisation of minimum ophthalmology competencies, developed collaboratively by the GMC, the RCOphth and medical schools, would support more consistent training across the UK, and future multi-institutional studies should evaluate whether such changes improve graduate competence and patient care.

## Appendices

Questionnaire with response options

1. Medical School

Options represented in the dataset:

UCL Medical School King's College London School of Medicine and Dentistry Leicester Medical School Anglia Ruskin University UCLan School of Medicine Manchester Medical School Barts and The London School of Medicine and Dentistry Imperial College School of Medicine Norwich Medical School University of Exeter Medical School Edge Hill Medical School University of Birmingham Medical School Southampton Medical School Sheffield Medical School Bristol Medical School St George's, University of London University of Cambridge Keele University School of Medicine Aston Medical School Liverpool Medical School Brighton and Sussex Medical School University of Nottingham Medical School University of Dundee, School of Medicine

2. Medical School/Medical Career Stage

Medical school/medical career stage as of 24/25

Options:

Year 1 Year 2 Year 3 Year 4 Year 5 Year 6 Intercalation FY1 FY2 Other

If Other, a free-text box was provided.

3. Referrer

If you were referred by someone to complete this survey, please enter their name below.

Response: Free text; apparently optional.

4. Gender

Options:

Male Female Prefer not to say Other

5. Year of Graduation

Year of graduation (Future or Past)

Response: Free text.

6. Ophthalmology Clinical Placement

Have you been offered (/will you be offered ophthalmology as a clinical placement in your medical undergraduate degree?

Options:

Yes No Unsure

Follow-up if Yes:

If yes, how long is the placement (in days)?

Response: Free text.

7. Ophthalmology SSC

Did you have the option of doing an ophthalmology-related student-selected component (SSC) at your medical school?

Options:

Yes No Unsure

8. Perceptions of Undergraduate Ophthalmology Education

To what extent do you agree with the following statements:

Statements:

My Medical school provides adequate teaching on ophthalmology. My Medical school provides adequate ophthalmology placement. Medical students at my university would benefit from more ophthalmology teaching and placement.

Five-point Likert response options:

Strongly disagree Disagree Neither disagree nor agree Agree Strongly agree

9. Additional Undergraduate Ophthalmology Experiences

What further undergraduate experiences do you feel would have been of most benefit to your education, regarding Ophthalmology? Please tick as many as you like:

Options:

Seminar (small, interactive group session) Lecture (large group session with little interaction) Informal clinic-based teaching Formal teaching with patients with Ophthalmology-related complaints Theatre time Other, please state

If Other, a free-text box was provided.

10. Perceptions of Ophthalmology and Ophthalmology Training

To what extent do you agree with the following:

Statements:

I am interested in surgery. I am aware of the portfolio requirements required to specialise in ophthalmology. Ophthalmology is a competitive field to specialise in. Ophthalmology training has a better work-life balance than other surgical specialties. Regarding gender identity, ophthalmology has a diverse workforce. Regarding racial identity, ophthalmology has a diverse workforce.

Five-point Likert response options:

Strongly disagree Disagree Neither disagree nor agree Agree Strongly agree

11. Specialty Interest

Medical/surgical speciality of interest

Response: Free text.

12. Self-Reported Ophthalmology Confidence

To what extent do you agree with the following:

Statements:

With my current knowledge, I can diagnose and treat common ophthalmic conditions. I can perform basic ophthalmoscopy and identify common abnormalities. I am confident in knowing when a referral to ophthalmology is needed.

Five-point Likert response options:

Strongly disagree Disagree Neither disagree nor agree Agree Strongly agree

13. Urgent Ophthalmology Referral Knowledge

Which of the following presentations do you believe would require urgent (within 24 hours) referral to the ophthalmology team (select all that apply)

The eight options were:

Experiencing sudden eye pain, halos around lights, and nausea is a concern Unexplained sudden loss of vision An intraocular pressure (IOP) greater than 40 mmHg Suspected Herpes zoster ophthalmicus Facial palsy with loss of corneal sensation Suspected temporal arteritis Papilloedema Sudden severe ocular pain
